# Bifid Uterus and Double Vagina

**Published:** 1866

**Authors:** 


### Bifid Uterus and Double Vagina.

The following interesting example of this malformation was communicated to the Boston Society for Medical Improvement (Aug. 28) by Dr. A. B. Hoyt, late Surgeon 25th Mass. Vols.:

The subject of it, aged 57 years, died of a cancerous tumor occupying the left illiac region. She had always been healthy until this disease made its appearance. There had been nothing unusul with regard to menstruation, which ceased at the age of 40. She had given birth to three children; her labors were always severe—the last one unusually so; this occurred twenty years before her death, and was achieved without instruments. The two previous confinements were protracted, but whether there was an abnormal presentation, or if instrumental aid was required, is not known. Her husband, during the patient's life, was ignorant of the fact that any unnatural condition existed. When pregnant, and whilst lactation was going on, menstruation was always suspended.

At the autopsy, the malformation was revealed for the first time, though there were some reasons for thinking that its existence had been known to the patient herself.

On examining the organ, it was found that there were two vaginæ, about equal in size, the left one perhaps a little the largest, and similar as to walls, rugæ, etc. They extended from just within the vulva to the uterus, and were separated by an interval filled with compact cellular tissue. Close to the uterus the vagina communicated with each other through an opening of about one fourth of an inch in diameter. From each vagina a probe passed into a separate uterine cavity. The os uteri in each vagina was small and imperfectly developed, as also was its orifice. The organ, as thus composed, was hardly larger than the normal uterus, but about one and a half inches from the os, it bifurcated into two symmetrical cornua, as large around as the forefinger, and about one and a half inches long; these terminated in the Fallopian tubes, which, with the ovaries and broad ligament, were natural. There was but one ovary to each cornu. The cornua were covered with peritoneum, except where the two layers of the broad ligament separated, and it also covered what might be called the fundus of the compound portion of the uterus. There was nothing to indicate that one side of the uterus had been impregnated and not the other, unless it was the greater capacity of the left vagina.—*Boston Med. and Surg. Jour.*, *Oct.* 26, 1865.

